# Conducting health survey research in a deep rural South African community: challenges and adaptive strategies

**DOI:** 10.1186/1478-4505-11-14

**Published:** 2013-04-24

**Authors:** Marisa Casale, Tyler Lane, Lebo Sello, Caroline Kuo, Lucie Cluver

**Affiliations:** 1 Health Economics and HIV and AIDS Research Division, University of KwaZulu-Natal, Private Bag X54001, Durban, 4000, South Africa; 2 Department of Psychology, University of Cape Town, Rondebosch, Cape Town, 7701, South Africa; 3 Department of Social Policy and Intervention, University of Oxford, Barnett House, 32 Wellington Square, Oxford, OX1 2ER, United Kingdom; 4 Department of Behavioral and Social Sciences and Alcohol Research Center on HIV, Brown University, Box G-S121-4, Providence, RI 02912, USA; 5 Department of Psychiatry and Mental Health, University of Cape Town, Rondebosch, Cape Town, 7701, South Africa

**Keywords:** Community research partnerships, Field research, Fieldwork challenges, Rural fieldwork

## Abstract

**Background:**

In many parts of the developing world, rural health requires focused policy attention, informed by reliable, representative health data. Yet there is surprisingly little published material to guide health researchers who face the unique set of hurdles associated with conducting field research in remote rural areas.

**Methods:**

In this paper we provide a detailed description of the key challenges encountered during health survey field research carried out in 2010 in a deep rural site in KwaZulu-Natal, South Africa. The aim of the field research was to collect data on the health of children aged 10 to 17 years old, and their primary adult caregivers, as part of a larger national health survey; the research was a collaboration between several South African and foreign universities, South African national government departments, and various NGO partners. In presenting each of the four fieldwork challenges encountered on this site, we describe the initial planning decisions made, the difficulties faced when implementing these in the field, and the adaptive strategies we used to respond to these challenges. We reflect on learnings of potential relevance for the research community.

**Results:**

Our four key fieldwork challenges were scarce research capacity, staff relocation tensions, logistical constraints, and difficulties related to community buy-in. Addressing each of these obstacles required timely assessment of the situation and adaptation of field plans, in collaboration with our local NGO partner. Adaptive strategies included a greater use of local knowledge; the adoption of tribal authority boundaries as the smallest geopolitical units for sampling; a creative developmental approach to capacity building; and planned, on-going engagement with multiple community representatives.

**Conclusions:**

We argue that in order to maintain high scientific standards of research and manage to ‘get the job done’ on the ground, it is necessary to respond to fieldwork challenges that arise as a cohesive team, with timely, locally-relevant, and often creative, solutions. Budgeting sufficient time and project resources for capacity building and community buy-in processes is also essential when working in remote communities unaccustomed to research. Documenting and sharing field experiences can provide valuable information for other researchers planning to conduct fieldwork in similar contexts.

## Background

In many parts of the developing world, rural health presents particular challenges that require focused policy attention. Rural areas are often characterised by worse population health outcomes and more difficult access to health care. In South Africa for example, the ten districts with the highest (material and social) deprivation indices are rural [1,2]. Contributing factors include poverty and lower education levels, limited services, geographic isolation and long distances to health services, as well as too few and under-resourced health facilities [2]. Many rural areas are also disproportionately affected by the HIV epidemic [2,3], partly because of AIDS-ill migrants returning to their rural homes to be cared for when they are no longer able to work in the cities [2,4]. Concerns around the status of rural public health have been a motivating factor in the development of partnerships among South African health practitioners and academic departments focusing on rural health^a^. Fortunately, in recent years there have been indications that the South African government, as well as other developing country governments, are assigning greater priority to rural health issues [5,6]. Still, a key concern remains that policies based on best practices for larger metropolitan centres are not always effective when implemented in rural contexts [5]. Understanding and addressing the particular health needs of rural areas requires, among other things, access to reliable and representative health data specific to these contexts.

However, conducting health survey research in remote rural areas comes with its own set of unique hurdles. In effect, the on-the-ground reality of conducting field research can be challenging in all settings, particularly in resource-scarce areas of the developing world [7-9]. However, working in isolated rural areas may be especially daunting, as a result of factors such as relative geographic isolation, limited services and distrust of outsiders. Unless addressed effectively, challenges that arise during the course of field research may threaten to delay and even jeopardise the overall success of data collection.

Yet, despite a growing appreciation of the value of cross-country collaborative health research between practitioners and academic departments, and the usefulness of documenting its challenges and lessons learnt [7,10-15], it is surprising how little published material health researchers will find, based on on-the-ground field experiences, to assist in informing and guiding their fieldwork plans and processes. For example, recently published journal articles documenting challenges of conducting field research in South Africa are very few and refer primarily to urban health research [7,8]. Research reports and publications often make scarce mention of the difficulties encountered during the process of collecting data, despite their potential value to the research community.

### Objective of this paper

The objective of this paper is to reflect on the process of conducting field research in resource-deprived HIV-endemic South African communities, in order to identify and discuss lessons that may be of value for future research carried out in similar settings. Specifically, we describe and discuss the four key challenges we experienced during field research conducted in 2010 in a rural site in northern KwaZulu-Natal, South Africa. In presenting each of the four challenges, we describe the initial planning decisions made, the difficulties encountered in trying to implement these and the strategies we adopted to respond to these difficulties. We conclude by reflecting on the observed outcomes of strategies adopted, as well as lessons learned that may be of value in informing future rural health field research.

The aim of the field research was to collect data on the health of children between the ages of 10 and 17, and their primary adult caregivers, as part of a national health survey conducted in three provinces. The primary objective of this research was to examine the impact of living in an AIDS-affected family on the physical, mental and social wellbeing of children and their caregivers, in order to identify risk and protective factors, and inform policy and programming. The research was a collaboration between the Health Economics and HIV and AIDS Division (HEARD) at the University of KwaZulu-Natal, the University of Oxford, Brown University and various South African government departments and non-governmental organisations (NGOs)^b^. Ethics approval was obtained from all partner universities and relevant government departments, and informed consent was obtained from all research participants.

The survey research in KwaZulu-Natal was conducted in two resource-deprived high HIV-prevalence sites: a rural site in the greater Manguzi area in the Umhlabuyalingana municipality, bordering southern Mozambique, and an urban site in Lamontville township within the eThekwini municipality. This paper focuses specifically on the experience of field research in the Manguzi field site, as many of the challenges and adaptive strategies were specific to its rural nature, thus providing unique learnings.

It should be noted that, in line with previous field process articles of this nature [7,8], this paper is a retrospective documentation of, and reflection on, a project fieldwork experience, on the part of the project management team (the authors are project managers or Principal Investigators (PIs)). It is therefore not based on a formal process of primary data analysis. However, the description of fieldwork experiences was, to a large extent, shaped by on-going collaboration and information exchange with the broader field research team and local NGO partner (as described in greater detail in the last paragraph of the Methods section). As a result of this regular communication and note-taking throughout the field research process, various sources documenting the rural field research process were available to draw from in developing this paper. These included project managers’ and PIs’ research notes and e-mail descriptions, as well as notes and minutes documenting regular team meetings with all (local and relocated) field research staff, and regular discussions with the NGO partner’s staff.

**Figure 1:**
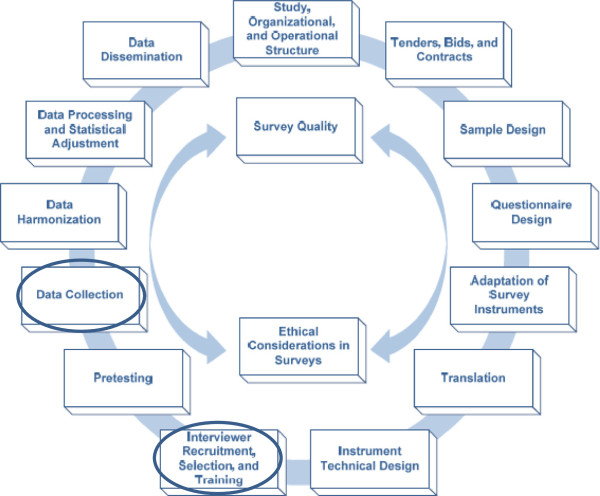
**The survey lifecycle.** Source [16].

Moreover, it is not our intention to attempt to provide an exhaustive list of steps to follow or potential factors to take into account at specific stages of the research project. Lengthy on-line resources are available to provide researchers with detailed guidelines in this regard [16]. Rather, the purpose of this paper is to share, with the broader research community, a detailed description of our most trying lived experiences, strategies and key lessons during our time in the Manguzi field site, pertaining to the field research preparation and implementation. These experiences are mainly relevant to two specific processes in the survey production lifecycle, highlighted in Figure 1[16], that is: ‘Interviewer recruitment, selection and training’ and ‘Data collection.’

The survey production lifecycle diagram, developed by the University of Michigan’s Survey Research Centre, represents all phases and aspects of the survey lifecycle, commencing with processes aimed at establishing study structure and ending with data dissemination [16]. It was developed to provide internationally recognised guidelines to highlight best practices for comparative survey research. As indicated in the diagram, quality and ethical considerations are relevant to all processes in this lifecycle. It is also noted that processes represented by the survey lifecycle need not be implemented in the exact order in which they appear in the diagram; there may be iteration within processes, and some of these processes may be simultaneous and interlocked [16]. For example, in the case of our survey, the survey instrument had already been finalised so that a standard instrument could be used in all national research sites; pre-testing of the tools included in the survey instrument had already occurred during pilot studies and previous studies prior to our relocation to Manguzi, and is therefore not discussed in this paper. The processes most relevant to the focus of this paper have been circled in the survey lifecycle diagram (Figure 1).

## Methods

### Description of the area

Manguzi is located in the Maputaland region of the KwaZulu-Natal province about 15 km south of the Mozambique-South Africa border. Its lack of road infrastructure (90% consists of sandy tracks) and transport, along with limited service availability and communication, make this community deep rural [17].

The Umhlabuyalingana municipality, in which Manguzi is located, spans an area of 3,621 km^2^ and was reported to have a population of approximately 164,000 people and an average household size of 6 people in 2007 [18,19], indicating an average population density of approximately 45 people or 7.5 households per km^2^. Ninety-nine percent of the municipality is classified as rural and about 60% of the municipal area falls under traditional authority ownership, while the remaining 40% constitutes commercial farms and conservation areas [19]. The population consists almost entirely of Black Africans (accounting for more than 99%) [19,20] and while the predominant language is Zulu, Tsonga is still widely spoken, especially among older inhabitants.

Available socio-demographic indicators highlight the high level of poverty and many social challenges in the area. In 2001, unemployment among the labour force was estimated to be around 70% [19,20]. According to the 2010/2011 Integrated Development Plan (IDP), 47% of the economically active population within the municipality receives either no income or less than R1600 (equivalent to approximately 180$) per month and only 8% of the population has obtained a grade 12 or higher education [19]. According to a 2008 report published by the South African Department of Social Development, more than half of the households in the greater Umkhanyakude District Municipality (incorporating Umhlabuyalingana and a further four municipalities) are female-headed, suggesting a high proportion of men having migrated from the area to seek employment [21]. Nearly 50% of all residents live in traditional dwellings [19], typically consisting of reed and/or thatch huts. While almost 50% of households have access to piped water, access to electricity and fixed line communication is still very low, with approximately 80% of the population dependent on energy sources such as candles and wood, and less than 1% accessing fixed communication lines (though about 70% of households have intermittent access to cellular telecommunication) [19]. The large majority of residents in the area travel by foot to reach schools, clinics and other destinations, and public transport is non-existent in many parts of the district [19,21]. Communicable diseases, including TB and HIV, accentuate health and social problems faced by communities in the greater Manguzi area [17] and life expectancy in the province is estimated to be 43 years [19].

### Description of our project and field research

Between 2009 and 2010 we conducted a household survey with 2,477 adult primary caregivers (18 years or older) and children aged 10 to 17 years old in their care, in the two (Manguzi and Lamontville) research sites. Sites were selected based on HIV prevalence rates (≥30% HIV prevalence among antenatal clinic attendees) [22], provincial health deprivation indices [23] and their respective representative urban and rural nature distinctions based on reasoning used in Statistics South Africa [24].

Research in Manguzi was carried out with the support of a well-established and respected local community-based non-governmental organisation (NGO), Tholulwazi Uzivikele (“Empower yourself through knowledge”), that had been working since the mid-1990s in the greater Manguzi area; the NGO’s activities included community based orphan care, early childhood and youth development, HIV prevention and community volunteerism [25]. We engaged with Tholulwazi Uzivikele because of the many known advantages of such community-academic collaborations. These collaborations are considered an important strategy for community health promotion, public health research and social problem-solving [14]; advantages include better access to community knowledge and awareness of the cultural and social context, the ability to use expertise in an applied manner, co-learning through knowledge exchange and greater potential for community acceptance and utilisation of findings [14,26-28]. We knew that our partnership with Tholulwazi Uzivikele would be crucial to granting the research team access to communities and legitimacy in the rural site, where households were unaccustomed to community-based health survey research. Moreover, we knew that their existing networks and contacts, and understanding of the local context, would be key to the success of the project.

Recruitment and training of field staff was carried out from March to April 2010, in collaboration with Tholulwazi Uzivikele, and the field research was conducted from May to October 2010. The rural field team consisted of two field coordinators (one local and one who had worked on the urban site and relocated from eThekwini to Manguzi), 14 interviewers (eight local and six from the urban field team who relocated) and five community guides. The decision to include relocated staff, who had previously worked on the urban site, was motivated by their good knowledge of the project and experience with the survey instrument, and the scarcity of local research skills. The intention was for the relocated staff to collaborate closely with local staff and assist with their capacity-building for the project. Three project managers coordinated the rural fieldwork process and the broader project; two of the project managers were from the local academic institution (HEARD at the University of KwaZulu-Natal) and one was from one of the overseas academic institutions (Oxford University).

The project managers rotated to ensure at least one project manager was present on field about 75% of the time; when on field they oversaw the field research logistics and rotated with interviewers to check the quality of interviews. Field coordinators provided supervision of the research process in the field by managing the research team when the project managers were not on site, identifying selected recruitment sites, driving interviewers and security escorts from meeting spots to interview sites, and regularly checking the quality of interviews and paper questionnaires. After obtaining consent from the local leaders and authorities (in this case the relevant tribal leaders), the interviewers approached every household in each designated area to ask a set of pre-defined consecutive screening questions in order to determine the household’s eligibility (*i.e.*, whether there was an adult caring for at least one child between the ages of 10 to 17). If households were identified as eligible and caregivers and children provided consent to participate, interviews were conducted, using standardized survey questionnaires with validated psychometric tools. The caregiver and child interviews were sequential, and caregivers were requested not to be present during the child interviews, in order to ensure confidentiality and minimize response bias. The community guides were individuals who had previously worked as home community-based carers within our NGO partner’s network; their main task was that of introducing the team and facilitating community buy-in. To ensure confidentiality, the community guides could not be present during interviews; after introducing the interviewers to a household, they would wait outside for the interview to be completed and/or move on to assist other interviewers. Given the high prevalence of HIV in these communities, it was particularly important to ensure the confidentiality of caregivers’ and children’s sensitive information, possibly including HIV status and/or HIV-related causes of household deaths, in order to protect these respondents from social stigma or other possible harm. No incentives were provided, but following the interviews participants received a certificate of thanks. Where there was a need for a particular participant to access more extensive support (such as seeing a counsellor or attending a clinic) referrals were made to the relevant social services offices . A referral system was set up, linked to the NGO partner organisation’s existing system, and all field staff were trained to deal with sensitive cases and referrals. Strict protocols were developed to regulate the quality control, hardcopy submission, transportation, and handover of questionnaires in field, as well as data storage. A total number of 1,279 pairs of interviews were completed on the rural site.

### Discussion and documentation of fieldwork progress, challenges and adaptive strategies

The regular field presence of project managers, daily conversations between field coordinators with both field staff and project managers, and frequent discussions between project managers and principal investigators meant that fieldwork progress and concerns could be quickly communicated among the project team. This also allowed urgent issues to be swiftly addressed, for example safety threats and ineffective field strategies. Moreover, project team meetings were held on a weekly basis; these meetings were facilitated by project managers, and field team members were encouraged to share anecdotes related to their field experience, raise concerns and relate difficulties experienced, as well as identify possible solutions. Similarly, the project managers held planning meetings with the NGO managers and staff on a regular basis, during which field progress, difficulties and adaptive strategies were also discussed. Notes were written up for all meetings. In addition, project managers regularly made handwritten and electronic notes on field experiences and reflections, as they were required to provide frequent updates of project progress, challenges and adaptive strategies to their respective research organisations and principal investigators.

## Results and discussion: key challenges and adaptive strategies

### Scarce existing research capacity

International best practice survey guidelines indicate that effective recruitment, selection and training of survey interviewers is essential in order to ensure good surve data quality [16]. Having the right field staff on board can minimize interviewer effects (measurement errors for which interviewers are responsible), while controlling costs by optimizing interviewer efficiency [16]. However, since no project of this nature had previously been conducted in Manguzi, and skills-building opportunities in the area were scarce, we knew it would not be possible to recruit local field team members who already had the appropriate research skills and experience for our project. At the same time, we wanted to work with a field team consisting predominantly of local staff, for various reasons: we knew their knowledge of the local culture and general context would add significant value to the project [28,29]; we knew it was important to the community that we train and hire young adults from Manguzi; and this was consistent with the ethos and commitment of all project partners to strengthening local research capacity as a means of advancing health and local development [15,28,30-32]. As indicated above, unemployment in the area was high and work options limited, so this study represented a unique opportunity for a selected group of individuals to gain field research training and experience that could help increase their future prospects of finding work. However, selecting staff without previous relevant experience – and in some cases no previous formal work experience at all – was a challenge. Moreover, we realised that we ran the risk of some individuals not managing to learn the required skills in the available time before the launch of the rural fieldwork.

Following discussion with our local NGO partner, we decided on a strategy that would both assist us in identifying potential fieldworkers and allow us to jointly build capacity. We chose to provide two weeks of research training to 28 individuals, representing twice the number of people that would be needed for our field team and the maximum number that the available venue could host. This training was to be seen as a capacity-building experience in itself, which we would acknowledge with a certificate of attendance. It would also represent an opportunity for us to interact with potential team members for two weeks and determine the best-suited individuals for our field work. The twenty-eight attendees were selected among individuals who had previously participated in a Youth Development programme with the NGO, based on a review of their CVs and the NGO’s feedback.

Project managers, one of the PIs and the urban field coordinator provided interactive training on research methods, research ethics, HIV/AIDS knowledge and questionnaire administration. Four days were dedicated to role play, during which each team member would take on the role of both interviewer and interviewee, thus allowing them the opportunity to practice administering consent forms and our project survey questionnaires in both English and Zulu. Particular attention was paid to administering psychometric scale questions correctly, by asking the questions exactly as written and correctly probing for most appropriate response options when participant responses were unclear. While language presented a barrier for some participants, trainees were responsive overall and eager to learn. On completion of the course, 14 field team members were selected by the project management team, based on both verbal and written assessments. Selection criteria included retention and understanding of the course’s content, spoken and written English and Zulu, attitude and personal interaction skills. In particular, community guides selected were individuals with previous home-based care experience through the local NGO partner, who were familiar with the local communities and demonstrated good personal interaction. In order to sensitively communicate our decisions as to who had and had not been selected, we met with each course participant individually; those who were not selected were given constructive feedback and encouragement, provided with ideas on how to develop further skills in certain areas, and promised that they would be contacted if opportunities arose in the future.

The selected field team members were then provided with a few days of further office-based training on the project research instruments, and were subsequently trained on-the-job. During the first month on field they were each partnered-up with a relocated experienced urban fieldworker who was familiar with the survey instruments; field coordinators and project managers rotated with fieldwork pairs to support individuals in building up skills and independence in the field. All field researchers were made to sign confidentiality agreements; however, in order to further protect confidentiality, local field interviewers were not permitted to conduct interviews, or be present during interviews, in their specific community. Fortunately, the local interviewers selected were from multiple communities so this did not pose a problem. Job profiles were drawn up and regular performance appraisals conducted with all staff, during which feedback was provided in five key areas: utilisation of training, adherence to protocols, team and community interactions, speed of work, and quality of work.

Despite our initial concerns, the performance of the selected Manguzi-based field interviewers exceeded all expectations, not least of all because of their determination and work ethic. Capacity building remained a priority for us throughout the project, and all field team members were provided with free opportunities to work with project managers on personal development plans and learn useful skills for their professional development beyond the research project (*e.g.*, personal development workshops, training in computer skills, CV writing and presentation skills provided by our project management team and the NGO staff).

Our experience reinforced how a lack of relevant local skills can be an obstacle in remote areas with little research history, and how this may require creative solutions. As indicated, all project partner organisations placed significant importance on local capacity building as key to quality improvement and individual personal development [28,30,31,33] and project funding was specifically allocated to these activities. It was particularly encouraging to observe individuals with no previous work experience become capable field researchers, and start to think a lot more about their future prospects. However, while we witnessed the benefits of developing capacity among local staff and working with a large diverse team, this required a considerable amount of time and energy dedicated to staff training and management of staff relations. Overall, our experience in Manguzi reinforced the importance of budgeting sufficient time and project resources at the project design stage, in order to maintain a developmental approach to staff’s personal development (*versus* simply focusing on project-related skills). Our experience also showed how teaming up with a local partner organization can be useful to highlight local skill gaps and availability, assist in designing, administering and hosting training, and help recruit potential trainees. Linking staff recruitment and performance management to a local community organization may also have the advantage of facilitating future job placement of the best-performing staff on termination of the field research.

### Staff tensions

Our initial concerns with hiring local field staff were mainly around their ability to learn the required skills for the project in a relatively short space of time, and their ability to work closely with the more experienced staff that had relocated from the urban site. However, as mentioned above, local staff performance did not prove to be a major obstacle, and initially nor did staff relations. Yet, unexpectedly, as more time passed, divisions and tensions started to form between different groups of staff. There were perceptions of some staff being considered or considering themselves superior to others and having authority over others; this was not only an issue between urban relocated and Manguzi-based field interviewers, but also between the community guides and local field interviewers. Tensions were likely worsened by the fact that urban staff members missed their home and their families in eThekwini, and experienced some initial difficulty in adapting to the new environment.

These perceptions of a hierarchy of importance among groups of staff members were very concerning to us as we cared a lot about team cohesion and all individuals feeling valued and equal. We believed this was central to staff morale and development, as well as their individual ability to use their specific skills and knowledge to add value to the project. We decided to address these issues on a number of levels, with the support of our NGO partner. We held individual discussions to determine the root of these tensions, used project meetings as a vehicle to communicate to staff the importance of the different and complementary skills that each staff member brought to the project, and also addressed staff tensions specifically with individuals where necessary. We held a facilitated discussion, during which groups of staff were encouraged to clarify misunderstandings and resolve any former issues honestly and openly. Our NGO partner organized an *ad hoc* personal development workshop that covered themes such as team work and professionalism, and together we provided various targeted training opportunities for all staff. We also organized team building activities, including day trips to a nature reserve in the area, during which we encouraged socialisation by ensuring a mix of gender and members from different staff ‘groups’ in each vehicle. Moreover, we were more careful to avoid referring to different staff groups using words such as ‘urban’ or ‘rural’, which we learned were unintentionally heightening divisions among staff. We also attempted to be more responsive to disrespectful behaviour as it arose and privately reprimanded individual team members where necessary. Lastly, we tried to improve the morale of relocated staff by providing them with an opportunity to return home every two weeks for short periods of time.

Though we recognise that the tensions that played out within our team derived in part from deeper-rooted sensitivities and stereotypes, which would not have been possible to resolve through one project, we do believe that the strategies employed were successful overall, as staff appeared to collaborate fairly well for the remaining duration of the project. Since many of these strategies were implemented at the same time, it is difficult to determine which of these may have been more or less successful. However, this experience taught us to not underestimate the challenges that may arise when forming a research team with both local and relocated staff, and to be more attentive to staff tensions that may manifest themselves later in the field research process. Lessons learnt include: explaining staff role profiles and highlighting the importance of each of these during initial training; covering themes such as professionalism and team interaction during this training (in conjunction with the local NGO partner); paying closer attention to staff interaction throughout the project, in order to pick up even subtle indications of possible tensions and address them swiftly; ensuring that at least some members of the field management team are fluent in the local language in order to follow verbal exchanges among staff members; avoiding language or terminology, in referring to various groups of staff, that may unintentionally reinforce stereotypes and staff division.

### Geographical and logistics constraints

Conducting fieldwork in the greater Manguzi area presented particular logistics challenges related to the area’s infrastructure and terrain. Sampling logistics and field plans needed to be adapted to take into account logistical constraints and the reality of political boundaries on the ground. For example, we had originally planned to use randomly selected census enumeration areas (EAs) in order to demarcate areas to be covered, and to use aerial photographs, maps and geographic information system (GIS) mapping as means to identify and locate each homestead. Our initial experience in field showed us that this would not be feasible in reality. Visibility was restricted in many areas due to flat terrain and dense bush; roads, road signage and/or road access to homes were absent in most areas. Aerial photos and maps were outdated and inadequate for our purposes. Reasons included: homogeneous terrain, without distinct landmarks to identify specific areas; footpaths (*versus* roads) leading from one homestead to another, which were often covered by vegetation and not visible in aerial photos; the fact that tribal community boundaries could not easily be geographically defined; and recent changes in the positions of homesteads and settlements (as many dwellings were constructed from reeds, stones and similar materials). While we continued to use GIS devices to map the location of homesteads visited, the absence of roads or GIS maps for the area made it very difficult to locate or relocate homes merely using GIS coordinates. In areas with poor visibility, field staff found it particularly challenging to identify homes and frequently became lost or disoriented, at times walking kilometres only to find homesteads that had already been visited by a fellow research assistant. Cell phone reception was also poor and non-existent in some areas, so communicating with the team or re-contacting respondents was difficult.

These challenges prompted quick re-assessment, in consultation with our local NGO partner, of both our household recruitment strategy in the rural site and the fieldwork logistics coordination. A decision was made to work within randomly selected *isigodi* or ‘tribal areas’, as opposed to census enumeration areas, as the smallest recognised geopolitical boundaries. Each *isigodi* was run by an *Induna*, a local tribal leader. Working with *isigodi* as units of stratification allowed us to refer to local knowledge and existing local political structures in order to determine community boundaries. Firstly, the census boundaries (wards and EAs) in Umhlabuyalingana were generally not recognised by communities as meaningful boundaries between communities, whereas *isigodi* were. Thus, while logistical factors such as dense bush coverage, out-of-date aerial photography maps, a lack of tarred ‘roads’ and a lack of landmarks made it very difficult to identify EA boundaries, *isigodi* boundaries, on the other hand, were recognised and could be identified by community members. Secondly, this demarcation made more sense for community buy-in, as tribal leaders were seen as the most important *de facto* authorities by community members, and their consent for us to work in their community was considered an important prerequisite for community participation.

Overall, a key strategy we used to address the logistics challenges on the rural site was to make use of local knowledge in various ways. For example, we worked with local drivers who were familiar with the area and terrain. We also developed a system by which our community guides would ‘scout’ each *isigodi* in conjunction with our NGO partner’s local network of home community-based carers present in that area, before the arrival of the field team; in doing so they familiarised themselves with the area and could subsequently direct interviewers to homes to avoid households being visited multiple times. Also, given the substantial distances between homesteads in certain areas, staff were frequently dropped off in different locations and later fetched by the same driver or field coordinator. In this way, local drivers and home community-based carer groups worked closely with our field team in each area to locate homes and direct interviewers. This system appeared to be successful, as the number of interviews conducted on a daily basis increased, and interviewers provided us with positive feedback on how this support had facilitated their work and reduced time wastage.

While we were aware that logistics and community buy-in in the rural area would be challenging, we realize in retrospect that it would not have been possible to fully ascertain and understand the level and nature of these challenges prior to starting the work on field. In less-accessible rural sites, which may be unfamiliar to researchers, it is important to allow for an initial period of time on field to test sampling logistics and field plans. Project management staff should to be prepared to go back to the drawing board, together with field staff and local partner organisations, in order to re-assess and refine these plans as early as possible, based on the logistics challenges and opportunities presented by the specific local context.

### Difficult community buy-in

Consulting with the community and obtaining the consent of key representatives to conduct research is essential for various ethical and practical reasons [34,35]. These include access to communities; trust and participation of community members; respect for community protocols, culture, and knowledge; greater accountability to communities; and safety of research team members [35-37]. We knew that obtaining consent from gate-keepers and the trust of community members would be especially important in a deep rural area such as Manguzi, where residents were unaccustomed to unknown visitors and to research, and overall diffident of outsiders unless introduced by their trusted leaders. Our experience during the early stages of fieldwork in Manguzi highlighted how difficult and stressful achieving this buy-in can be [9]. Following discussion with our NGO partner, we agreed that the NGO’s staff would take the lead in community buy-in activities. This decision was a reasoned one, based on the trust they had gained in the community, their understanding of the political structures and the cultural environment [9], and our concern around creating undue expectations of material gain among community leaders [7,38]. The NGO staff followed all the correct processes, including presenting the project at tribal leaders’ meetings and obtaining consent from the main tribal chief in the area.

Nevertheless, it was apparent that knowledge of the project had not reached all relevant communities and/or households upon our arrival. As a result, we encountered much distrust and many refusals, and in extreme cases were chased out of communities and even labelled ‘Satanists’. We are still not clear as to the origin of these accusations, but believe they were simply a result of misunderstandings around the nature of research, and fear and distrust toward outsiders (as our local research staff reported anecdotes of children having disappeared from schools and a general fear of Satanistic activities). It should also be noted that our fieldwork in Manguzi was launched only a few months before the commencement of the 2010 soccer World Cup in South Africa, and we learned that communities had been warned about the dangers of child trafficking.

We also later learnt why many households were not aware of our project. Not all community leaders had attended tribal meetings and/or advised their community members about the project, nor had all community members attended meetings, and communication could not happen quickly as distances between households were substantial. Moreover, some communities were temporarily without tribal leaders due to succession processes in course.

We decided to rethink and intensify our community dissemination, in close collaboration with our NGO partner. Our team liaised with the NGO’s staff to immediately contact the local schools and the *Induna* from each community in order to explain the purpose of our research and how this would be carried out. Consequently, plans were made to meet each *Induna* individually and obtain consent to work within his area of authority. The consultation process ended up involving multiple meetings with both local leaders and communities, during which these stakeholders would have the opportunity to ask for further clarification regarding our project. These meetings had to be planned well in advance, in order to take into account the tribal leaders’ availability and dates of planned community meetings. We realized that it was important for both NGO partner representatives and research team members to be present at community meetings, since community leaders and members wanted to engage with individuals working for a trusted community organisation, but also wanted to receive clarification on questions related to the research project directly from research team members. Moreover, community guides and home community-based carers used their ‘scouting’ activities as an opportunity to verbally introduce the project, disseminate flyers and provide any additional information requested. The fact that this information was provided by known members of the community was important to potential participants, as it meant that it could be trusted. Overall the strategies adopted to address community buy-in appeared to be extremely successful, as they significantly improved the reception of our team members by communities, and reduced the number of refusals to a negligible amount.

Through this experience we learnt the extent to which community buy-in in deep rural areas can be a lengthy and cumbersome process [9], possibly extending throughout the duration of the project and requiring numerous interactions with multiple gatekeepers. When planning field research in remote rural areas it is important to take into account the resources (including management time) needed for these activities, and the potential delays in commencement of fieldwork in certain communities, where tracking down gatekeepers and obtaining their approval may prove difficult. In our case, community buy-in proved most successful when working closely and consistently with our local NGO partner’s staff, and approaching local leaders and communities together. While working with a respected local partner is key, the best approach to community buy-in will to some extent depend on the specific local context.

## Conclusions

No matter the extent of scientific rigour, consultation and planning, unexpected obstacles will inevitably emerge during field research, and these will require adaptive strategies to be overcome. In particular, conducting survey research in remote rural areas of the developing world may pose unique challenges that require appropriate context-relevant responses. Unfortunately, text books and the current body of scientific papers do little to equip researchers for the experience that awaits them. Obtaining trust and buy-in from key gatekeepers, overcoming logistics difficulties, effectively developing local skills and managing staff relocation are some fieldwork aspects that may be particularly trying when working in deep rural settings.

Our experience is testimony to the importance of responding in a timely manner to fieldwork challenges as they emerge, and reconciling the tensions that may arise in goals of producing high quality scientific research with the practical feasibility of ‘getting the job done’. Effectively responding to obstacles encountered on the field is not simply about reaching target numbers of participants and meeting deadlines, it is also about ensuring good quality data. For example, poorly trained and/or unmotivated project staff can result in refusals and item non-response, and reduce the reliability of data collected, while the inability to access a large proportion of identified communities and/or households can lead to sampling bias [16]. We believe that overall our adaptive strategies were successful, since we did not encounter any major safety incident, maintained a healthy relationship with our local partner, managed to obtain consent to work in most communities, and met our research objectives within the expected timeframe. When we returned to the researched communities in 2011 in order to provide initial community feedback to stakeholders and participants in collaboration with our local NGO partner, no particular challenges arose and overall our visits and feedback were well received.

However, as discussed above, this experience taught us many practical lessons that we will take forward for future field research design and implementation. It also demonstrated the value of a large, cohesive research team and regular communication among partners and project team members. In particular, we realised the importance of a strong local partnership, based on mutual respect, clear agreed goals of collaboration and shared development interests [26,27], but also the willingness and honesty to question decisions and discuss alternative approaches. Moreover, the frequent field visits of our PIs, the significant presence of project managers on field, and regular discussions among the project management team and the NGO, allowed us to identify threats as they emerged and critically re-assess and refine fieldwork plans within a short space of time.

In brief, our story of a field research experience in rural South Africa is one of many difficulties, but also, we believe, of overall success. It was an invaluable experience, which has provided us not only with important data, but also with stronger partner networks and useful lessons. Our findings, currently in the process of being analysed and written up, highlight the (physical and mental) health risks among caregivers and vulnerable children living in poor, HIV-endemic South African communities, reinforcing the importance of this type of work with similar populations. Moreover, our on-going experience in managing field research continues to teach us that doing good research is not about having a problem-free experience (should such a thing exist), but rather about being ready and able to deal with challenges that arise, in a timely and effective manner. This entails finding feasible – and often creative – solutions that work in a given local context, while maintaining the scientific integrity of the study. Lastly, we believe that sharing these field experiences and our responses to them can provide valuable information for other researchers planning to conduct similar work in similar contexts. As such we hope that the volume of published articles in this area will increase.

## Endnotes

^a^ For example, the Rural Health Advocacy project is a partnership between the Wits Centre for Rural Health, the Rural Doctors Association of Southern Africa (RuDASA) and SECTION27, including the AIDS Law Project; their website is: http://www.rhap.org.za. The University of KwaZulu-Natal’s Centre for Rural Health has also been active in providing policy feedback and support: http://crh.ukzn.ac.za/Home.aspx. ^b^ Further information on both the KwaZulu-Natal and national survey research project is available on the following website: http://www.youngcarers.org.za

## Abbreviations

GIS: Geographic information system; NGO: Non-governmental organization; PI: Principal investigator.

## Competing interest

The authors declare that they have no competing interest.

## Authors’ contributions

All authors made substantial contributions to the conception and design, acquisition of data, and analysis and interpretation. LC and CK made a greater contribution to the conception and design, while MC, LS and TL made a greater contribution to the acquisition of data. MC took the lead role in drafting the manuscript, and all other authors contributed to this process and critically revised the manuscript. All authors read and approved the final manuscript.

## Authors’ information

MC is a researcher at the Health Economics and HIV and AIDS Research Division at the University of KwaZulu-Natal, and a doctoral candidate in Psychology at the University of Cape Town. TL is a doctoral candidate at the Department of Social Policy and Intervention at the University of Oxford. LS is currently Program Manager for U.S. Peace Corps South Africa. CK is a postdoctoral fellow in Child and Adolescent Biobehavioral HIV at Brown University. LC is a lecturer at Oxford University and the University of Cape Town. MC, TL and LS were Co-Project Managers for the KwaZulu-Natal component of the Young Carers South Africa project. CK and LC were Co-Principal Investigators of the Young Carers South Africa project.
